# Functional characterization of the transient receptor potential melastatin 2 (TRPM2) cation channel from *Nematostella vectensis* reconstituted into lipid bilayer

**DOI:** 10.1038/s41598-023-38640-6

**Published:** 2023-07-15

**Authors:** Andras Szollosi, János Almássy

**Affiliations:** 1 Department of Biochemistry, Semmelweis University, Tuzolto u. 37-47, Budapest, 1094 Hungary; 2 Department of Physiology, Semmelweis University, Tuzolto u. 37-47, Budapest, 1094 Hungary; 3 ELKH-SE Ion Channel Research Group, Semmelweis University, Tuzolto u. 37-47, Budapest, 1094 Hungary; 4 HCEMM-SE Molecular Channelopathies Research Group, Semmelweis University, Tuzolto u. 37-47, Budapest, 1094 Hungary

**Keywords:** Permeation and transport, Ion channels, Ligand-gated ion channels

## Abstract

Transient receptor potential melastatin 2 (TRPM2) cation channel activity is required for insulin secretion, immune cell activation and body heat control. Channel activation upon oxidative stress is involved in the pathology of stroke and neurodegenerative disorders. Cytosolic Ca^2+^, ADP-ribose (ADPR) and phosphatidylinositol-4,5-bisphosphate (PIP_2_) are the obligate activators of the channel. Several TRPM2 cryo-EM structures have been resolved to date, yet functionality of the purified protein has not been tested. Here we reconstituted overexpressed and purified TRPM2 from *Nematostella vectensis* (nvTRPM2) into lipid bilayers and found that the protein is fully functional. Consistent with the observations in native membranes, nvTRPM2 in lipid bilayers is co-activated by cytosolic Ca^2+^ and either ADPR or ADPR-2′-phosphate (ADPRP). The physiological metabolite ADPRP has a higher apparent affinity than ADPR. In lipid bilayers nvTRPM2 displays a large linear unitary conductance, its open probability (P_o_) shows little voltage dependence and is stable over several minutes. P_o_ is high without addition of exogenous PIP_2_, but is largely blunted by treatment with poly-l-Lysine, a polycation that masks PIP_2_ headgroups. These results indicate that PIP_2_ or some other activating phosphoinositol lipid co-purifies with nvTRPM2, suggesting a high PIP_2_ binding affinity of nvTRPM2 under physiological conditions.

## Introduction

TRPM2 is a non-selective cation channel which belongs to the largest family of TRP channels, the melastatin-like TRPM family which counts eight known members^1,2^. TRPM2 is widely expressed in several organs^3–5^ and underlies important physiological processes such as insulin secretion by pancreatic beta-cells^6^, monocyte chemokine production^7^, and regulation of body temperature by neurons of the hypothalamus^8,9^. TRPM2 activation is also observed under pathological conditions including chronic neurodegenerative disorders and neuronal cell death induced by reactive oxygen species^3,10–14^.

TRPM2 is a homotetrameric protein, which consists of an ~ 800 amino acid N-terminal and a ~ 400 residue C-terminal cytosolic region that flank the transmembrane domain (TMD). The TMD is built by six transmembrane helices (S1-6) and embraces a voltage-sensor like domain (VSLD, S1-4) and the pore domain (PD, S5-6). The PD consists of an extracellular non-selective cation permeable selectivity filter and a cytoplasmic gate formed by the S6 helix-bundle. TRPM2 also comprises a unique ~ 270 residue C-terminal extension, which shares ~ 40% sequence homology with the mitochondrial ADPR hydrolase (ADPRase) enzyme NUDT9^5^, and is therefore called the NUDT9-homology (NUDT9-H) domain.

TRPM2 is directly activated by cytosolic Ca^2+^^15,16^ and ADP-ribose (ADPR) binding^3,5,17^, however, phosphatidylinositol-4,5-bisphosphate (PIP_2_) in the membrane is also indispensable for channel activity^18^. Invertebrate TRPM2 channels hydrolyze their bound ADPR ligand, while ADPRase activity is absent in vertebrates^19^. Loss of enzymatic activity in vertebrate channels coincided with the appearance of a rapid pore collapse mechanism^18^, termed rundown.

Several invertebrate and vertebrate TRPM2 structures have been resolved to date^20–25^ that promoted our understanding of TRPM2 function. The structures revealed that the selectivity filter of TRPM2 is wide and short compared to that of a K^+^ channel^26^, explaining the lack of ion selectivity^25^. In contrast to family members TRPM4 and 5^27,28^, the pore of TRPM2 is also permeable to divalent cations, likely due to the observed subtle differences between their filters^2,25^. Both the cytosolic domains and the TMDs are involved in ligand binding. While Ca^2+^ is coordinated by side chains of S2–S3 residues at the membrane-cytosol interface, ADPR binds to two distinct sites. One is formed by the TRPM-homology regions (MHR) of the N-terminal domain^20,21^, the other is found in the NUDT9-H domain^20^. The functional relevance of dual ADPR binding is, however, not yet fully understood and shows strong cross-species variations. ADPR bound to the MHR site is visible in the structure of both the zebrafish (drTRPM2) and the human (hsTRPM2) channel, while a ligand-occupied NUDT9-H site was observed only in the latter structure^1,20,21^.

All structural information on TRPM2 has been obtained from overexpressed, purified, detergent solubilized protein. But whether those preparations contain functionally active protein has not been tested. Moreover, for many ion channels auxiliary subunits strongly contribute to functional properties in living cells, shaping their responsiveness to various activating stimuli^29–33^. Although for TRPM2 channels binding sites for all known ligands are formed by the pore-forming subunits, and so far no auxiliary subunits have been discovered, it is important to verify to what extent the functional properties of purified TRPM2 channels resemble those of TRPM2 currents recorded from intact cells, or from patches of native cell membrane.

We have previously purified the TRPM2 protein from *Nematostella vectensis* (nvTRPM2) and determined its structure in detergent micelles^25^. We also characterized the enzymatic activity of nvTRPM2^19^. Robust ADPRase activity confirmed that the NUDT9-H domains are functional. We have also reported that ADPR gates the channel independently of its hydrolysis^19^, and that the ADPR analog, ADPR-2′-phosphate (ADPRP), a physiological metabolite, is also a full agonist of nvTRPM2 with a higher apparent affinity compared to ADPR^34–36^. On the other hand, in the solved structure the pore is closed, and currently no data is available to support that the purified protein forms a functional ion channel capable of gating. Here we reconstituted the nvTRPM2 protein into planar lipid bilayers and tested its functionality to verify whether responsiveness to its known ligands resembles that reported for nvTRPM2 in native cell membranes.

## Results and discussion

### Expression and purification of nvTRPM2

**Figure 1:**
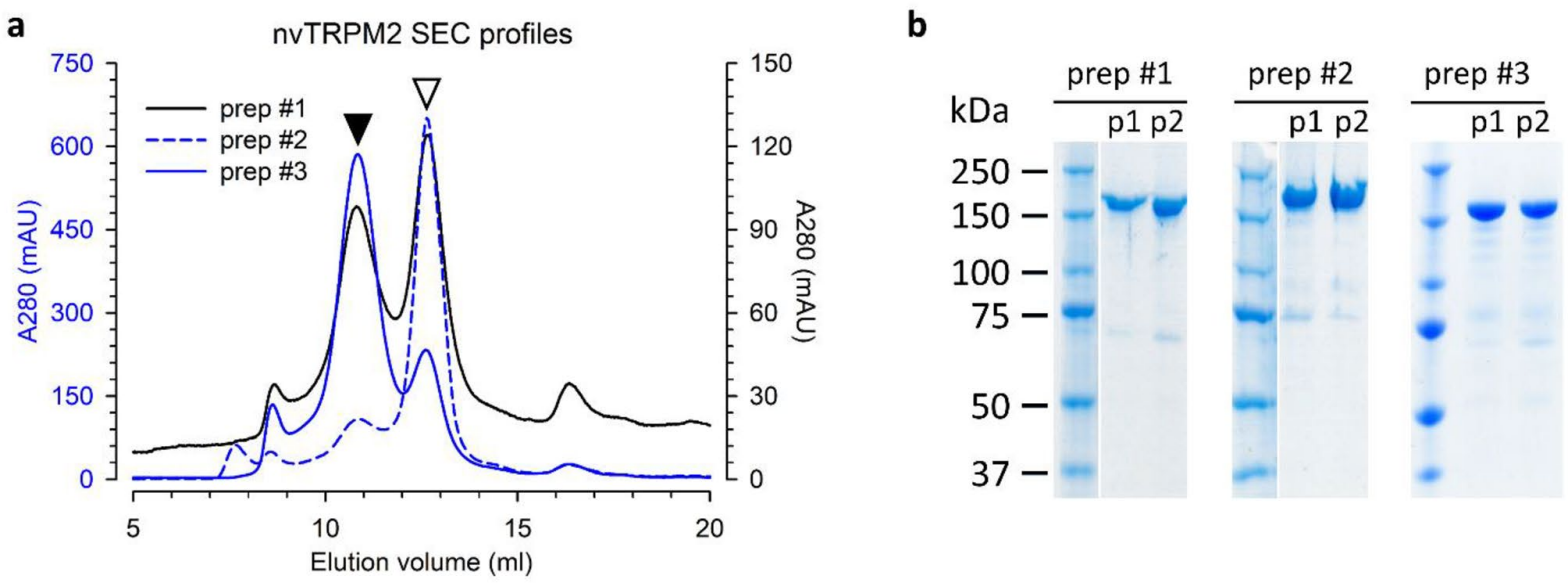
Purification of nvTRPM2. (**a**) Size-exclusion chromatography (SEC) profile of the nvTRPM2 protein from three different preparations used for reconstitution. Protein eluted in two consecutive peaks (at 10.7 and 12.6 mls, *filled and open arrows*, respectively). In preparations 1 (*solid black*) and 3 (*solid blue*) the peak 1 fraction was abundant. Protein from preparation 2 (*dashed blue*) mainly accumulated in peak 2. SEC profiles of preparations 2 and 3 are plotted with a separate A280 axis (left blue). (**b)** SDS PAGE image of collected peak 1 and 2 fractions (p1 and p2) from the three preparations in (**a**). Gel pictures displaying purity of p1 and p2 fractions are cropped images. White vertical line separates molecular weight standard from sample bands in preparations 1 and 2. Original gels are presented in Supplementary Fig. 1.

nvTRPM2 was expressed using the BacMAM expression system^37^. Protein pre-purified by affinity chromatography was further purified by size-exclusion chromatography (see “Methods” Section). nvTRPM2 eluted from the Superose 6 column as two consecutive peaks at 10.7 and 12.6 ml (p1 and p2) (Fig. 1a). The relative abundance of the protein in the two peaks varied from one preparation to another. Pooled fractions corresponding to individual elution peaks were kept separately from each other and concentrated to 1.5–2.4 mg/ml. The purified ~ 176 kDa protein was clean and mostly free from contaminations, and single bands from the two separate peaks looked similar on 7.5% SDS-PAGE gels (Fig. 1b and Supplementary Fig. S1), suggesting that the posttranslational modification pattern of the protein in p1 and p2 is similar. Both p1 and p2 fractions were used for the bilayer experiments, and basic gating characteristics of the channel proteins obtained from the two distinct elution peaks were similar in functional measurements. We, therefore, speculate that the two peak fractions correspond to different oligomeric states of the TRPM2 protein, yet upon the ~ 500 × dilution of the sample into the bilayer chamber the p1 oligomers disassemble and allow single channel recording upon reconstitution.

### Reconstituted nvTRPM2 is functional and is activated by Ca^2+^ and ADPR

In the first line of experiments we confirmed that nvTRPM2 protein reconstituted into bilayers, in symmetrical 140 mM NaCl containing solutions, is also activated by Ca^2+^ and ADPR. Effects of Ca^2+^ chelation and of addition of incremental concentrations of nucleotides (ADPR or ADPRP) were tested at -80 mV. In contrast to the unstable pore of hsTRPM2^18^, nvTRPM2 channels show no rundown in inside-out patch clamp experiments^25^. Similarly, nvTRPM2 activity remained stable for several minutes following reconstitution into lipid bilayers.

**Figure 2:**
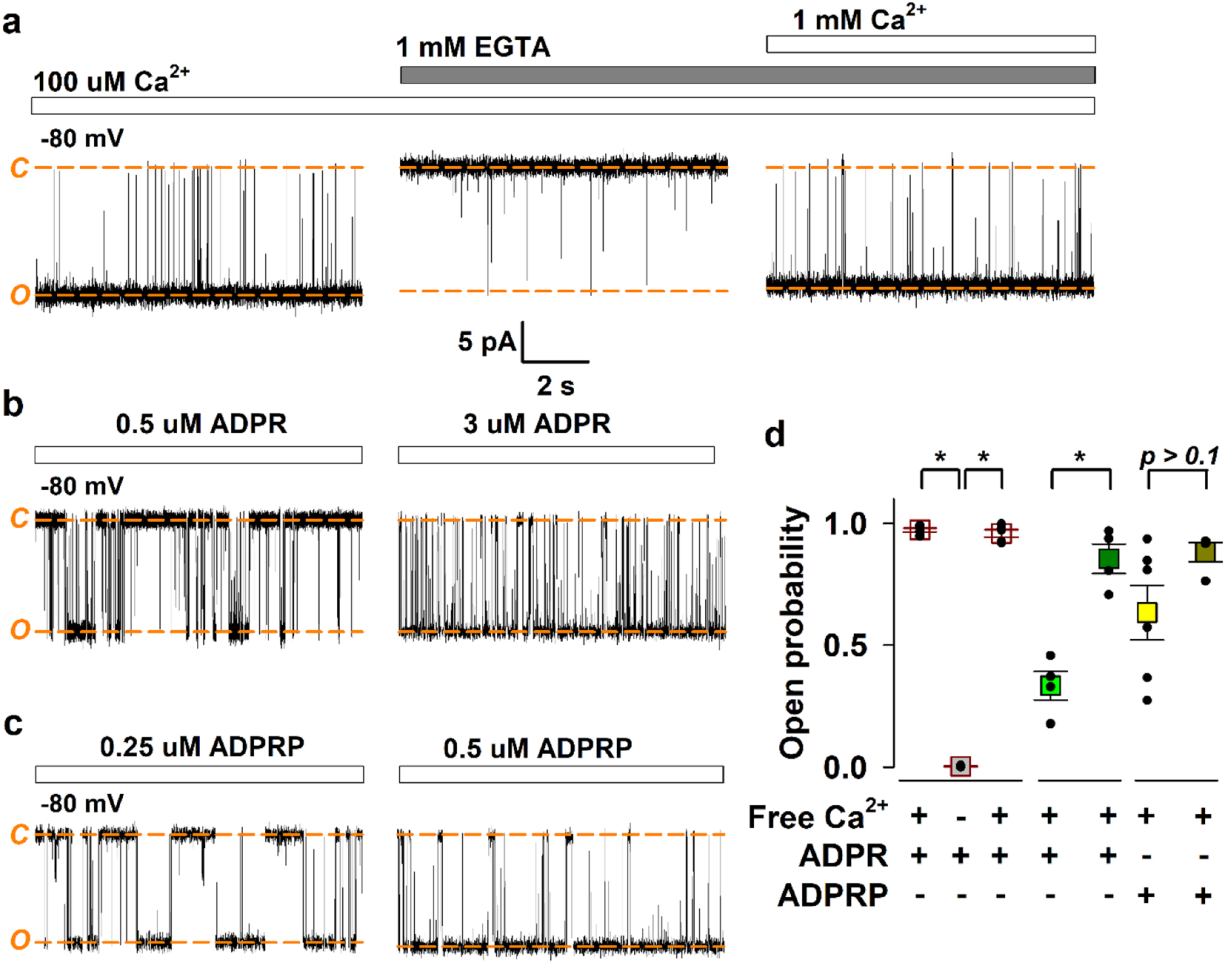
nvTRPM2 reconstituted into lipid bilayers is co-activated by Ca^2+^ and ADPR(P). (**a**) Effect of Ca^2+^ chelation by EGTA on channel activity shown for a representative trace. Displayed segments are parts of the same continuous recording, current levels of open (*O*) and closed (*C*) channels are indicated with *orange dash*, L-bar indicates time-scale and current amplitudes for (**a**–**c**). P_o_ values of analyzed segments (mean ± S.E.M., n = 5) are summarized in (**d**). (**b**) Effect of ADPR concentration on channel activity shown for a representative trace. P_o_ values of analyzed segments (mean ± S.E.M., n = 4) are summarized in (**d**). (**c)** Effect of ADPRP concentration on channel activity shown for a representative trace. P_o_ values of analyzed segments (mean ± S.E.M., n = 4–6) are summarized in (**d**). (**d**) Dot plot summary of P_o_ values obtained in (**a**–**c**). Mean ± S.E.M. P_o_ values were compared by Student’s unpaired *t*-test to assess statistical significance (*p < 0.01).

At -80 mV downward deflections reflect channel opening. In the presence of 100 μM Ca^2+^ and 50 μM ADPR in the cis (cytosolic) compartment open probability (P_o_) was high (0.973 ± 0.008, n = 5) (Fig. 2a left and Fig. 2d). Upon addition of 1 mM EGTA to the cis compartment, thus in the nominal absence of Ca^2+^, channel activity declined to near zero (P_o_ = 0.003 ± 0.002, n = 5), only infrequent brief openings were detected (Fig. 2a middle and Fig. 2d). Reintroducing Ca^2+^ by overtitrating EGTA restored channel activity to its original level (P_o_ = 0.959 ± 0.015, n = 5) (Fig. 2a right and Fig. 2d). Therefore, just as in intact cell membranes, nvTRPM2 gating in lipid bilayers is strictly Ca^2+^-dependent, channel activity in ADPR alone is negligible.

In inside-out patch-clamp experiments ADPR and its derivative ADPRP are both full agonists of the nvTRPM2 channel, and the apparent affinity of ADPRP is ~ fivefold higher^36^. Here we tested the effects of both nucleotides on nvTRPM2 gating in lipid bilayers. Currents of reconstituted channels were first recorded in the presence of 0.5 μM ADPR in the cis buffer, in the presence of 100 μM Ca^2+^ (Fig. 2b left). Increasing the ADPR concentration to 3 μM (Fig. 2b right) caused a significant increase in channel P_o_, from 0.334 ± 0.059 (n = 4) to 0.855 ± 0.061 (n = 4) (Fig. 2d). Thus, ADPR is a stimulatory ligand for the purified nvTRPM2 protein, and our limited data set suggests an EC_50_ in the range of ~ 1 μM, in good agreement with previous estimates (~ 2 μM^19,36^). Channel P_o_ in 0.25 μM ADPRP was 0.634 ± 0.111 (n = 6) (Fig. 2c left and Fig. 2d), and was further increased, although statistically not significantly, by raising ADPRP concentration to 0.5 μM (0.882 ± 0.040, n = 4) (Fig. 2c right and Fig. 2d). Channel P_o_ was significantly higher (*t*-test *p* = 0.0002) in 0.5 μM ADPRP compared to that in 0.5 μM ADPR, thus ADPRP is a higher-apparent affinity agonist of purified nvTRPM2: its estimated EC_50_ of ~ 0.2 μM is consistent with that determined in patch-clamp experiments (0.4 μM^36^).

In summary, when reconstituted into lipid bilayers the purified nvTRPM2 protein, whose structure was previously resolved by cryo-EM^25^, remains a Ca^2+^-sensitive channel co-activated by either ADPR or ADPRP, and the latter nucleotide is a higher-apparent affinity agonist.

### nvTRPM2 in planar lipid bilayers is mildly voltage sensitive

**Figure 3:**
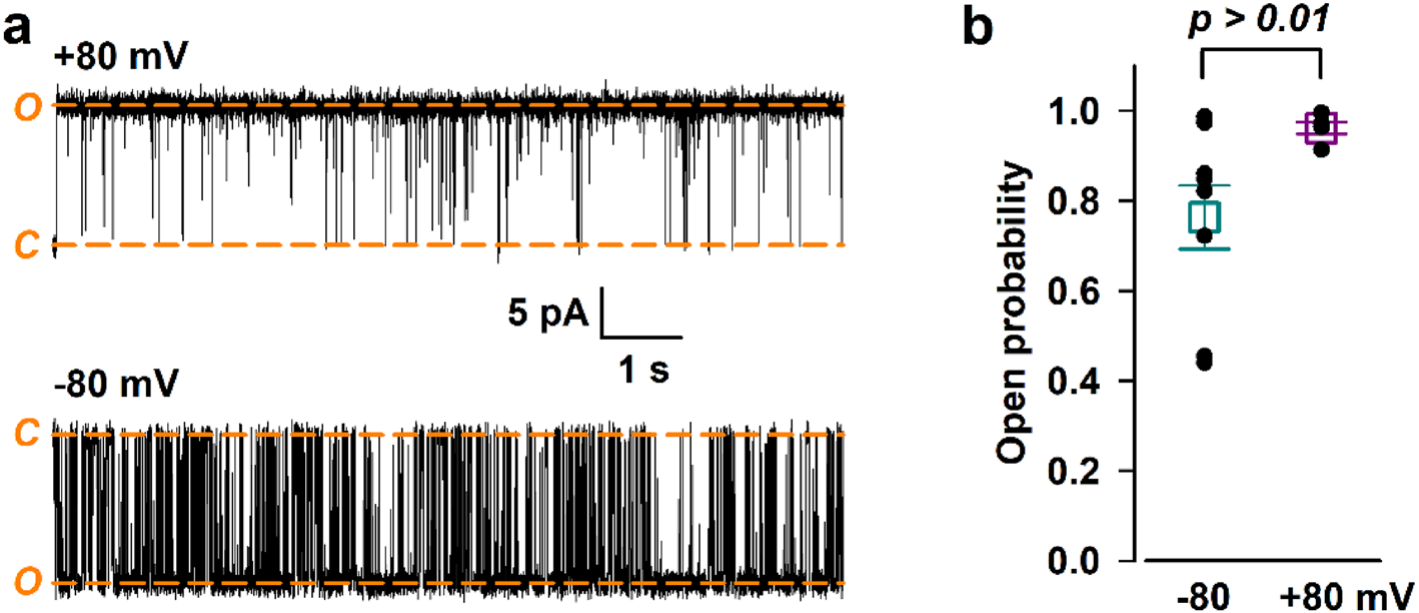
P_o_ of nvTRPM2 in lipid bilayers is little voltage-sensitive. (**a**) Representative traces recorded at + 80 and − 80 mV. At + 80 mV upward, while at − 80 mV downward deflections reflect channel openings under our bilayer conditions. Displayed segments are parts of the same continuous recording, current levels of open (*O*) and closed (*C*) channels are indicated with *orange dash*, L-bar indicates time-scale and current amplitudes. (**b**) Dot plot summary of P_o_ values (mean ± S.E.M., n = 5–9). Difference between data is not statistically significant.

The TMD region of TRPM2 resembles that of voltage-gated cation channels^38,39^. While in the latter conserved positively charged residues in S4 serve as the gating charge, nvTRPM2 lacks most basic S4 residues in its VSLD and hence is reportedly little voltage sensitive in inside-out patch-clamp experiments^25^. To assess voltage sensitivity of nvTRPM2 activity in lipid bilayers, we determined P_o_ of the channel at − 80 and + 80 mV (Fig. 3a). We included only single-channel measurements in the analysis which precludes uncertainty in the total number of active channels in the bilayer. Similarly to previous reports, P_o_ of nvTRPM2 was little voltage sensitive (0.76 ± 0.07, n = 9 at − 80 mV vs 0.96 ± 0.01, n = 5 at + 80 mV; *t*-test *p* = 0.064) (Fig. 3b) in planar lipid bilayers. The obtained P_o_ at − 80 mV is very close to the value of ~ 0.8 measured for nvTRPM2 at − 20 mV in inside-out patch-clamp experiments with or without Mg^2+^^19^.

### Addition of exogenous PIP2 is not required for activity of purified nvTRPM2 in lipid bilayers

**Figure 4:**
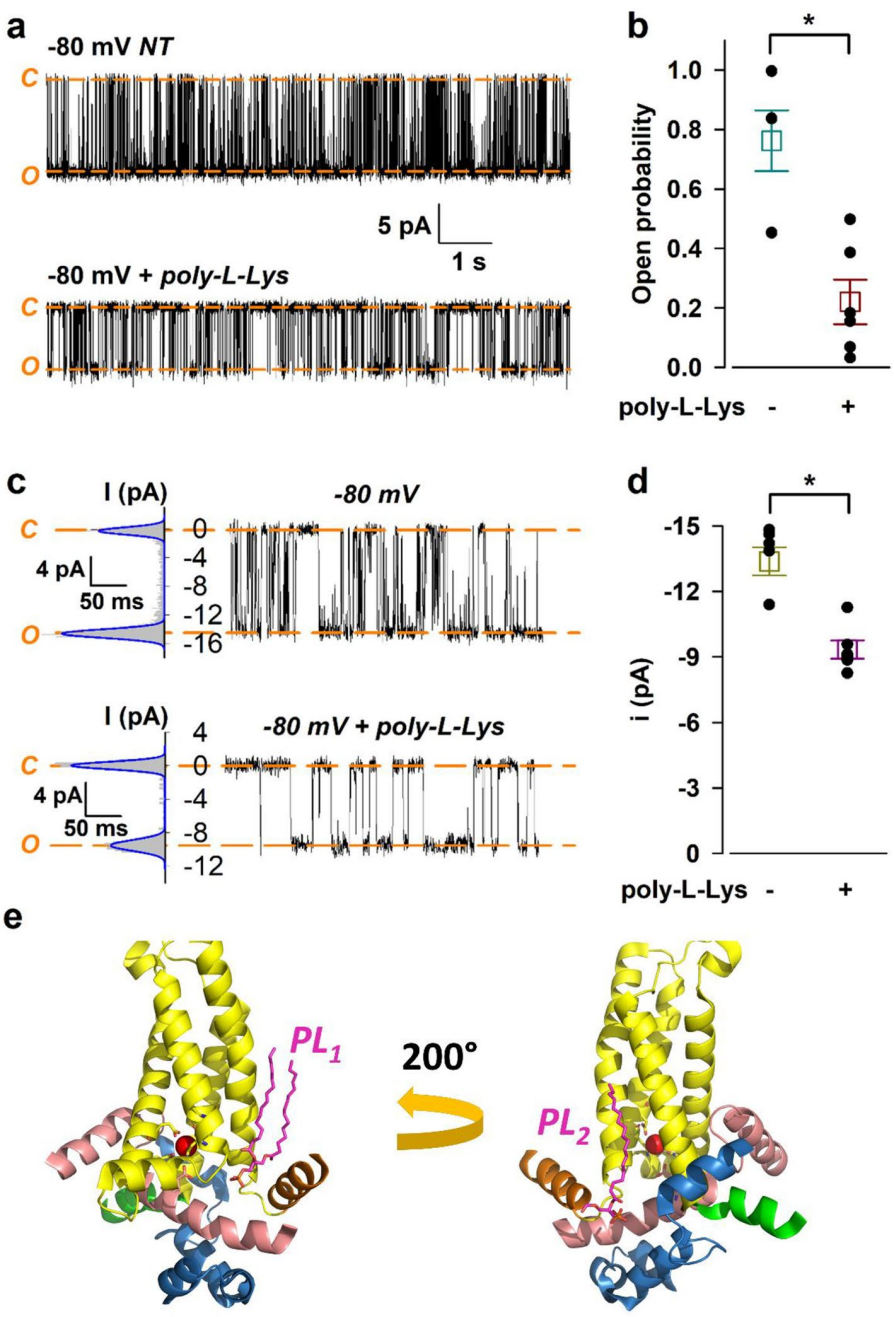
Effects of poly-l-Lysine on nvTRPM2 steady-state single channel currents. (**a**) Effect of 15 μg/ml poly-l-Lysine on channel activity shown for a representative trace recorded at − 80 mV. Downward deflections reflect channel openings under our bilayer conditions. Displayed segments are parts of the same continuous recording, current levels of open (*O*) and closed (*C*) channels are indicated with *orange dash*, L-bar indicates time-scale and current amplitudes. (**b**) Dot plot summary of P_o_ values (mean ± S.E.M., n = 6). (**c**) Effect of 15 μg/ml poly-l-Lysine on unitary current amplitude. Displayed segments are parts of the same continuous recording, current levels of open (*O*) and closed (*C*) channels are indicated with *orange dash*, L-bar indicates time-scale and current amplitudes. Fits of sums of Gaussian functions (*blue*) to all-points histograms (*gray*) are shown to the left of representative current traces recorded at − 80 mV. (**d**) Dot plot summary of *i* values (mean ± S.E.M., n = 6). *i* was calculated as the distance between Gaussian peaks in (**c**). Mean ± S.E.M. P_o_ values were compared by Student’s unpaired *t*-test to assess statistical significance (*p < 0.01) in (**b**) and (**d**). (**e**) Phospholipid (PL_1_ and PL_2_, *magenta sticks*) binding to the vanilloid-type (*left*) and the TRPM3- and TRPM8-like site (*right*) in nvTRPM2 (6co7). Selected parts of the cytosolic MHR domain (*blue*), the pre-S1 helix (*green*), VSLD (S1-4, *yellow*), the S4–S5 linker (*orange*) and the TRP 1–2 helices (*salmon*) are colored. Residues on the S2, S3 and TRP 1 helices that coordinate the Ca^2+^ ion (*red sphere*) are shown as sticks with Pymol element coloring.

A P_o_ value close to unity at + 80 mV (Fig. 3b) is an interesting observation, considering that the third obligate agonist, PIP_2_, was not added to the chamber and was also not included in the bilayer lipid mixture. One explanation for the observed lack of effect of PIP_2_ is that in the planar bilayer membrane nvTRPM2 does not require PIP_2_ binding for activity. An alternative explanation is that PIP_2_ is bound to the channel with high affinity even subsequent to detergent-extraction of the channel from the native membranes. To test the later possibility, we employed poly-l-Lysine, a polyamine cation that weakens the channel-PIP_2_ interaction by masking the negative headgroups of the phospholipid^40^. 15 μg/ml poly- l-Lysine markedly lowered the P_o_ of nvTRPM2 channels in lipid bilayers (from 0.76 ± 0.10, n = 6 to 0.22 ± 0.08, n = 6; *p* = 0.0016) (Fig. 4a–b) suggesting that PIP_2_ remains bound to nvTRPM2 throughout the protein purification procedure. Currents were recorded at − 80 mV, since at positive voltages the polycation caused a complete block of channel activity. Even at the used negative membrane potential the high dose of poly-l-Lysine significantly reduced the amplitude of the unitary channel current (from − 13.38 ± 0.64 pA, n = 6 to − 9.34 ± 0.42 pA, n = 6; *p* = 0.0004), as expected for voltage-dependent pore-block by a polycation (Fig. 4c–d).

High-affinity PIP_2_ binding to nvTRPM2 is further supported by several earlier findings. On the one hand, while in the presence of saturating Ca^2+^ and ADPR poly-l-Lysine closes nvTRPM2 channels in native membrane patches, addition of exogenous PIP_2_ to untreated channels causes only modest (10–20%) further stimulation^25^. This implies that the low endogenous concentration of PIP_2_ present in inside-out patches is sufficient to support close-to-maximal currents, suggesting that the ligand is tightly bound to the protein. On the other hand, in the cryo-EM structure of apo nvTRPM2 a phospholipid, potentially PIP_2_, is visible in the vanilloid binding pocket, in close vicinity to the Ca^2+^-binding site^25^ (Fig. 4e left, PL_1_). The protein used for that structural study was prepared identically as described here (the LMNG detergent micelle was also replaced by digitonin during preparation), and yet the phospholipid remained bound to the protein all the way until it was loaded on grids. While the vanilloid site is found to bind phosphatidic acid or PIP_2_ also in other TRP^41,42^ or K^+^ channels^43^, it is not the only PIP_2_ binding site observed in structures. A phospholipid bound to a cleft on the opposite side of the VSLD, surrounded by parts of the MHR domain, the pre-S1 elbow and the TRP helices, is also seen in nvTRPM2^25^ (Fig. 4e right, PL_2_), TRPM3^44,45^, TRPM8^46^, and Drosophila NOMPC^47^ channels. Mutational analysis identified the latter region as the primary PIP_2_ binding site in human and nvTRPM2 channels^48^. The same study also showed that TRPM2 activity—abolished by scavenging of endogenous phospholipids with poly- l-Lysine—can be partially restored also by other phosphoinositol lipids (PI(3,4)P_2_ or PI(3,4,5)P_3_). Either way, a tight interaction between PIP_2_—or potentially other phospholipids—and nvTRPM2 could explain the high P_o_ observed in bilayer measurements.

### Unitary current amplitudes depend linearly on membrane voltage

**Figure 5:**
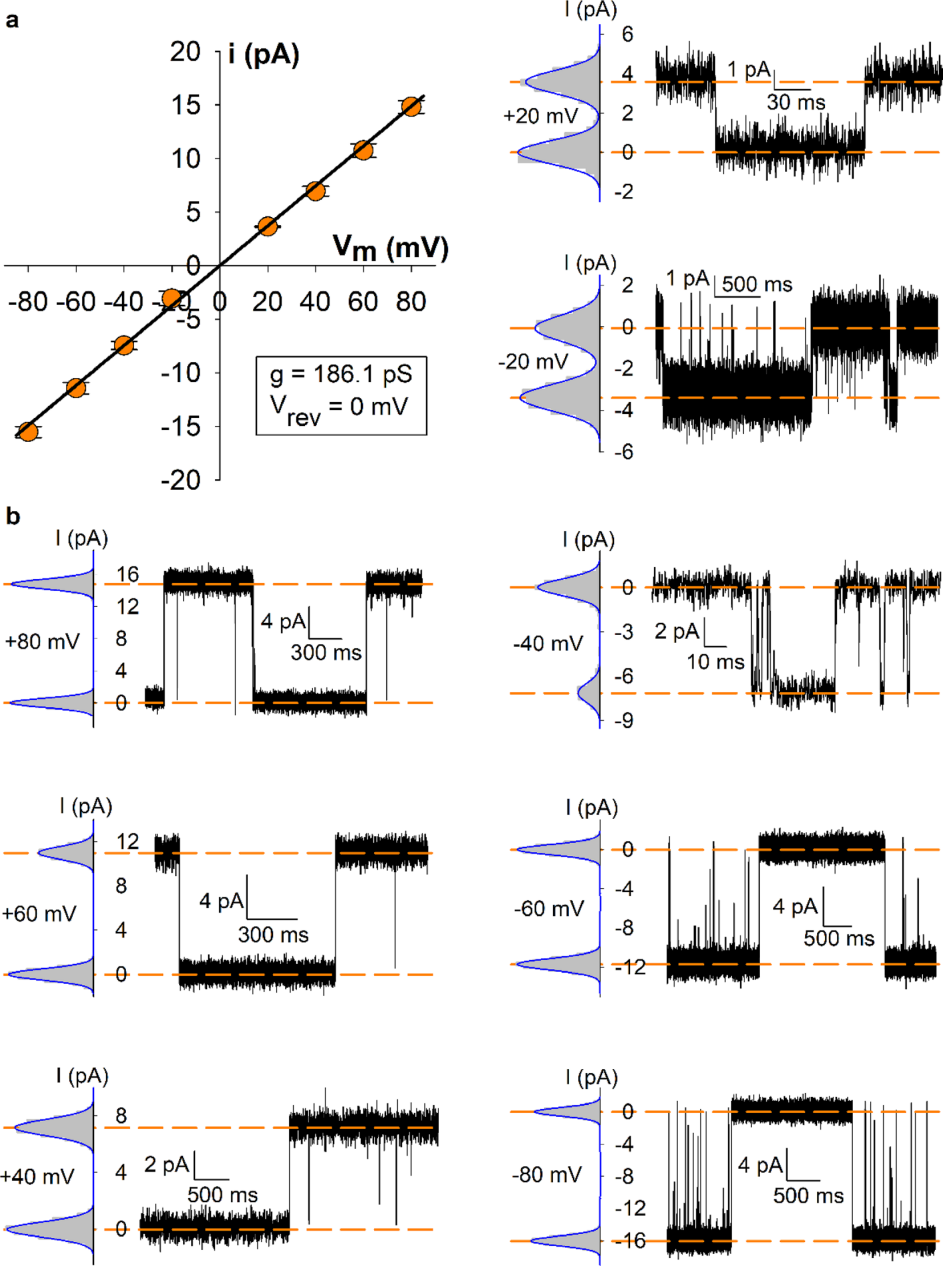
Unitary conductance properties of nvTRPM2. (**a**) Unitary current amplitude-voltage (*i*–V) relationship of nvTRPM2 in symmetrical 140 mM Na^+^ as the main charge carrier, in the presence of 100 μM Ca^2+^ in the cis (cytosolic) compartment. Symbols (*orange circles*) represent *i* (mean ± S.E.M., n = 3–5). Unitary conductance was calculated by linear regression (*black line*). (**b**) Fits of sums of Gaussian functions (*blue*) to all-points histograms (*gray*) are shown to the left of each current trace. *i* was calculated as the distance between Gaussian peaks (marked with *orange dashed lines*) at the indicated membrane potentials. L-bars indicate time-scale and current amplitudes for traces.

To assess unitary conductance of nvTRPM2 in lipid bilayers, we estimated unitary current amplitudes in the range of − 80 and + 80 mV membrane voltages (Fig. 5). Plotting unitary current amplitudes (*i*) as a function of membrane voltage reported a linear *i*–V relationship (Fig. 5a). As expected for symmetrical 140 mM NaCl solutions, the reversal potential was ~ 0 mV. Unitary conductance calculated from the slope of the current–voltage plot was 186 pS (R^2^ of linear regression = 0.998).

These features align well with the conductance properties of nvTRPM2 in inside-out patches. A non-linear *i*–V relationship with a conductance of ~ 150 pS was reported for nvTRPM2 under conditions where both in- and outside solutions contained 2 mM Mg^2+^, which causes pore block^25^. However, when Mg^2+^ was omitted from all solutions, the *i*–V relationship became linear and unitary conductance was > 200 pS^19^, a value comparable to that we obtained here in bilayer measurements, under similar conditions. Of note, cytosolic Ca^2+^ at the 100 μM concentration employed here for channel stimulation causes only very minor pore block in inside-out patch-clamp experiments^36^.

### Conclusions

TRPM2 is a non-selective cation channel, the function of which was assessed in several whole-cell and cell-free studies. nvTRPM2 is an invertebrate homologue of human TRPM2. The structure and ADPRase activity of nvTRPM2 was previously reported, but gating of the purified protein has never been tested yet. Here we show that the functional properties of nvTRPM2 reconstituted into planar lipid bilayers closely resemble those of nvTRPM2 in native cell membranes, thus, these established functional properties are intrinsic to the TRPM2 protein itself. As observed in inside-out patch-clamp experiments, the reconstituted channel is co-activated by Ca^2+^ and either ADPR or ADPRP, and the latter nucleotide is a higher-apparent affinity agonist. Its unitary conductance is large and linear. In the presence of saturating agonists its P_o_ is close to unity, especially at positive voltages. The high P_o_ is obtained without addition of external PIP_2_, but is markedly reduced upon scavenging by poly-l-Lysine treatment, suggesting that some activating phospholipid is bound tightly to the protein, and is not washed out during the purification process. Based on our experiments we cannot distinguish whether that lipid is PI(4,5)P_2_ or some other activating phosphoinositide. Nevertheless, these findings suggest a high phospholipid binding affinity of the channel under physiological conditions.

## Material and methods

### Materials

All chemicals were purchased from Merck if not indicated otherwise.

### TRPM2 purification

The *Nematostella vectensis* TRPM2 (nvTRPM2) protein was expressed and purified as discussed previously^25^. In brief, the pRML-13 BacMam expression vector (generous gift from Eric Gouaux) harboring the coding sequence of nvTRPM2 with a C-terminal GFP tag was transformed into DH10 Bac cells (Invitrogen). The isolated bacmid was transfected into Sf9 cells (ATCC) and recombinant baculoviruses were generated in three infection cycles (P1-3). P3 virus was transduced into HEK 293S GnTI^-^ cells (ATCC), and following overnight incubation protein expression was induced by 10 mM sodium butyrate at 30 °C for 48 h^37^.

For protein purification, harvested HEK cells were resuspended in lysis buffer (50 mM Tris–HCl pH 8.0, 2 mM MgCl_2_, 200 mM NaCl, 20% Glycerol, and 1 mM DTT) supplemented with protease inhibitors. nvTRPM2 protein was extracted from membranes by addition of 1% 2,2-dodecylpropane-1,3-bis-β-D-maltopyranoside (LMNG) (Anatrace) and 0.1% cholesteryl hemisuccinate (CHS) (Anatrace). Protein was pre-purified by affinity chromatography using anti-GFP nanobody (His-tag-purified from *E. coli* lysates) immobilized to NHS-Sepharose (Cytiva). While the protein was bound to the column, detergent in micelles was exchanged to digitonin by extensive wash in Buffer A (20 mM Tris–HCl pH 8.0, 150 mM NaCl, 0.06% digitonin, and 1 mM DTT). Protein was eluted by GFP-tag cleavage using PreScission protease (Cytiva). The eluate was further purified by size-exclusion chromatography in Buffer A on a Superose 6 10/300 column (Cytiva).

### TRPM2 reconstitution and single channel recording

Purified nvTRPM2 was incorporated into planar lipid bilayers. Phosphatidylethanolamine, phosphatidylserine, and phosphatidylcholine (Avanti Polar Lipids) lipids were mixed at a ratio of 5:4:1. The dried mixture was resuspended in n-decane at a final concentration of 20 mg/mL. Lipid membranes were painted on the 200 um diameter aperture of a Delrin cuvette fixed between cis and trans compartments of a BCH-M13 chamber (Warner Instruments). Bilayer formation was monitored by currents measured between sample (cis) and reference (trans) electrodes attached to the headstage of an Axopatch 200B amplifier (Molecular Devices) using an episodic 5 mV voltage pulse protocol. nvTRPM2 protein diluted in Buffer A was injected into the cis compartment beyond the aperture. Channel currents at variable membrane potentials were amplified using an Axopatch 200 amplifier and recorded using pCLAMP 9.2 (Molecular Devices) software. Currents were digitized at 10 kHz and filtered at 1 kHz by an eight-pole low-pass Bessel filter. Buffer composition of the chamber allowed selective detection of channels with identical orientations in all experiments. Symmetrical buffer B (20 mM TRIS–HCl pH8.0; 140 mM NaCl) on both sides was supplemented with 100 μM CaCl_2_ and 50 μM ADPR (except for dose–response measurements where ligand concentrations are indicated) in the cis compartment, but with 1 mM EGTA in the trans compartment. Hence, only TRPM2 channels with the cytosolic side facing the cis compartment were activated. Only measurements with a single nvTRPM2 channel reconstituted into the bilayer were used for analysis. ADPRP (Biolog) and poly-l-lysine were added to the cis compartment, where indicated.

### Statistics and analysis

All data are presented as mean ± S.E.M. Statistical significance between data groups was calculated by unpaired two-tailed *t*-test using GraphPad online. Difference between data was considered statistically significant for *p* < 0.01.

Open probability (P_o_) was determined from single-channel measurements. Current traces were baseline subtracted and idealized using half-amplitude threshold crossing. Open and closed dwell times were collected into an eventlist and open probability was calculated as the cumulative open time divided by the total recording time.

Unitary current amplitudes were estimated by fitting Gaussian functions to all points-histograms of single-channel traces, and defined as the distance between adjacent peaks.

Structure figures were generated using Pymol (http://www.pymol.org).

## Supplementary Information

Supplementary Figure S1.

## Data Availability

Data will be made available on request. Any request should be addressed to the corresponding author at szollo5@gmail.com.
