# Supplementary material for: Functional characterization of the transient receptor potential melastatin 2 (TRPM2) cation channel from Nematostella vectensis reconstituted into lipid bilayer
Source: Sci Rep. 2023 Jul 15;13:11471. doi: 10.1038/s41598-023-38640-6 (PMC10349829; doi:10.1038/s41598-023-38640-6)
Supplement: Supplementary file 1 — Supplementary Figure S1. [file 41598_2023_38640_MOESM1_ESM.docx]

**
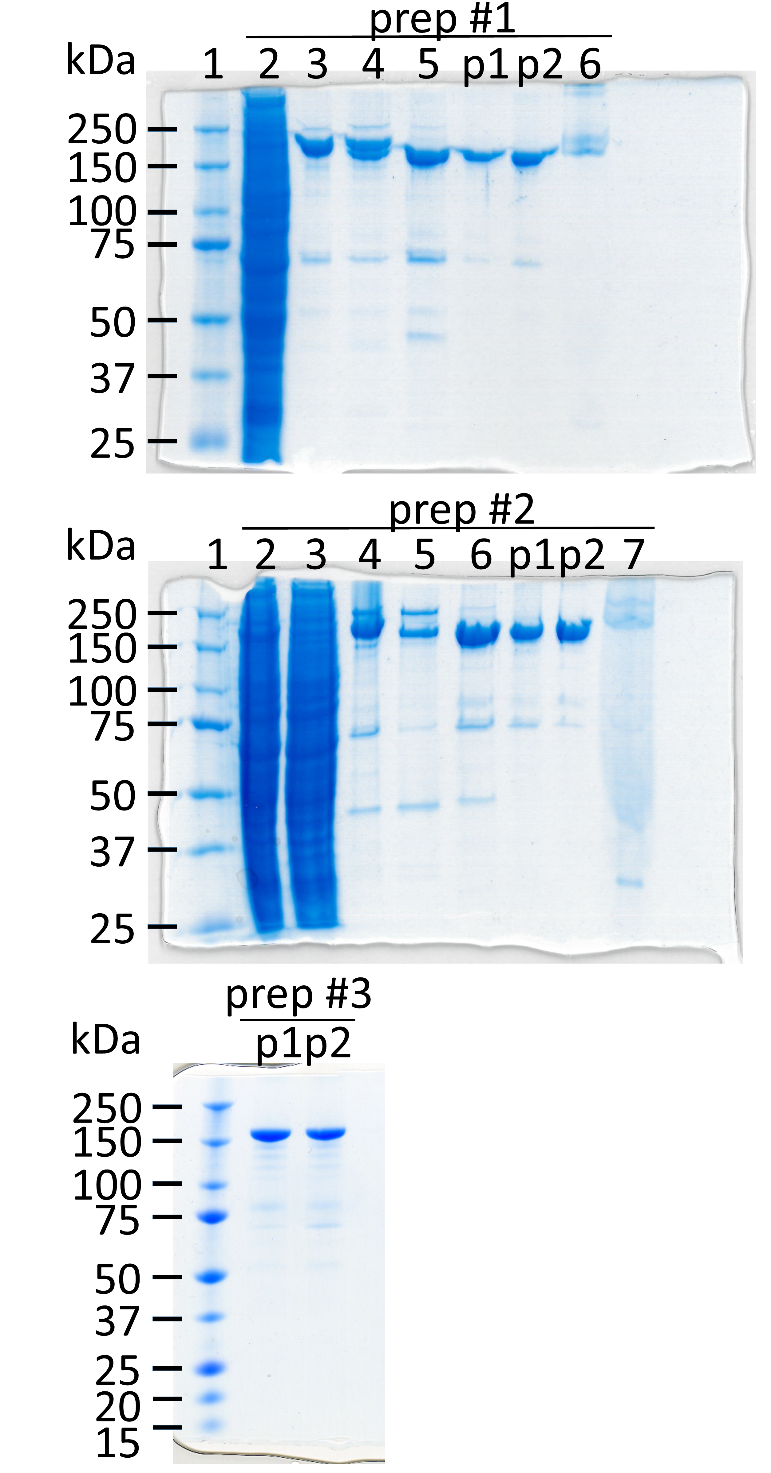
Supplementary Information**

**Figure S1.** Uncropped, original gel images from Figure 1.B left to right, respectively. Fractions during purification are loaded on 7.5% SDS polyacrylamide gels. *Top* in order from 1 to 6: molecular weight standard, unbound affinity fraction, affinity resin before tag cleavage, affinity resin after tag cleavage, concentrated fraction prior to size exclusion chromatography (SEC), p1 SEC fraction, p2 SEC fraction, guanidine-HCl eluate of affinity resin; *Middle* in order from 1 to 7: molecular weight standard, lysate supernatant, unbound affinity fraction, affinity resin before tag cleavage, affinity resin after tag cleavage, concentrated fraction prior to size exclusion chromatography (SEC), p1 SEC fraction, p2 SEC fraction, guanidine-HCl eluate of affinity resin. *Bottom* molecular weight standard, p1 SEC fraction, p2 SEC fraction
